# Understanding alpha-synuclein aggregation propensity in animals and humans

**DOI:** 10.1016/j.bbrep.2024.101810

**Published:** 2024-08-14

**Authors:** Natalie G. Horgan, Annie M. McCarty, Ashley A. Hetak, Hailey B. Penticoff, Jessica S. Fortin

**Affiliations:** a Department of Basic Medical Sciences, College of Veterinary Medicine, Purdue University, West Lafayette, IN, 47906, United States; b Department of Pathobiology and Diagnostic Investigation, College of Veterinary Medicine, Michigan State University, East Lansing, MI, USA, 48824, United States

**Keywords:** Aggregation, Alpha-synuclein, Fibril, Parkinson's disease, Thioflavin T (ThT), Transmission electron microscopy (TEM)

## Abstract

Alpha-synuclein (α-syn) aggregation plays a critical role in the pathogenicity of Parkinson's Disease (PD). This study aims to evaluate the aggregation propensity of α-syn fragment peptides designed using the variability found in humans and animals. Thioflavin T (ThT) and transmission electron microscopy (TEM) were used to validate the formation of fibrils to identify important amino acid residues. Human α-syn fragments 51–75, 37–61, 62–86, 76–100, and 116–140 demonstrate a significantly higher tendency to aggregate compared to fragments 1–25, 26–50, and 91–115. All species analyzed of the α-syn 37–61 and 62–86 regions were shown to form fibrils on both ThT and TEM. The α-syn 37–61 and 62–86 fragment regions exhibited a high susceptibility to aggregation, with fibril formation observed in all species. The A53T mutation in several α-syn 37–61 fragments may enhance their propensity for aggregation, suggesting a correlation between this mutation and the capacity for fibril formation. Furthermore, the presence of the non-amyloid-β component (NAC) region, specifically in α-syn 62–86, was consistently observed in several fragments that displayed fibril formation, indicating a potential correlation between the NAC region and the process of fibril formation in α-syn. Finally, the combination of a high quantity of valine and a low quantity of acidic amino acids in these fragments may serve as indicators of α-syn fibril formation.

## Highlights

• The presence of the NAC region was consistently observed in all the fragments that displayed fibril formation.
• The 37–61 and 62–86 peptide fragments exhibited a high susceptibility to aggregation.
• High quantity of valine and low quantity of acidic amino acids in these fragments may serve as indicators of alpha-synuclein fibril formation.

## 1 Introduction

Alpha-synuclein (α-syn), along with Beta- and Gamma-Synucleins, are strongly expressed in neurons [1]. Of the three, α-syn is best known due to its relationship to neurodegeneration; the other two synucleins are less studied [2]. α-syn has been found to have 140 amino acid residues, split into 3 regions: the N-terminal, the NAC (non-amyloid-β component) domain, and the C-terminal. These regions perform different functions that contribute to α-syn's ability to aggregate and spread throughout the brain. The N-terminal (1-60) binds lipids and adopts α-helical structure, which is essential in regulating the protein's interaction with cellular membranes. The NAC domain (61–95) contributes to α-syn's self-propagation and binds the N- and C-terminals. The C-terminal (96–140) is responsible for interactions with other entities such as proteins and small molecules within the CNS tissue to maintain solubility and regulate α-syn aggregation [3]. The exact function of α-syn is not completely elucidated, though it is thought to be involved in numerous signaling pathways and the regulation of the neurotransmitter dopamine, which plays a crucial role in regulating fine motor control and other physiological functions [4,5].

When α-syn misfolds, it will eventually aggregate and form inclusions within the neurons called Lewy bodies, which are a hallmark of synucleinopathies [4]. One of the most well-known synucleinopathies is Parkinson's Disease (PD), a neurodegenerative disease resulting from the deterioration of dopaminergic neurons located in the substantia nigra [6]. Patients with PD may exhibit a wide range of clinical symptoms, including (but not limited to) bradykinesia, rigidity, involuntary tremors, and cognitive degeneration [5,7]. These symptoms are the result of Lewy body formation within the neurons. The accumulation of Lewy bodies results in cell death, upon which they are released into the extracellular space. Healthy neurons will then intercept the recently liberated extracellular α-syn and further propagate through fibril seeding [8]. Eventually, misfolded α-syn will exceed the brain's clearance threshold, leading to widespread inflammation and neurodegeneration [3]. In this way, α-syn spreads through neural tissue like prion diseases such as bovine spongiform encephalitis [4]. α-Syn has been associated with additional neurodegenerative disorders. In multiple system atrophy, α-syn involves non-neuronal cells, such as glial cytoplasmic inclusions [9]. There have been 5-point mutations linked with the development of early onset PD: A30P, E46K, H50Q, G51D, and A53T [[10], [11], [12], [13], [14], [15], [16]]. These mutations are within the N-terminal domain, which is the most highly conserved fragment of α-syn across species [1]. These mutations provide α-syn with multiple methods of conformation, directly affecting α-syn's functional properties [17].

The native conformations of α-syn are the monomer and tetramer forms—depending on the protein's location and interactions with its surrounding cells [18]. The fibrillization of α-syn, a process vital for the advancement of α-synucleinopathies, perpetuates the propagation, resulting in the fibril formation that induce severe motor impairment and dopaminergic neuronal death [18]. Lewy Bodies are rich in α-syn fibrils. This fibrillar form is found to be toxic to neurons through a variety of mechanisms, including chronic inflammation and perturbing cellular ion homeostasis [17]. Further, the oligomerization of α-syn constructs, even shorter ones, is effectively facilitated by the product ONE, which results from the peroxidation of polyunsaturated fatty acids that are present withing the neuronal cell membrane [19]. In its toxic oligomeric form, α-syn is known to rupture cellular membranes [20]. Oligomeric α-syn conformations are also seen to be more toxic than their fibrillar counterparts, as they have been shown to perturb the lipid bilayer and mitochondrial functions of the cell [21]. There are 4 main strategies to combat the pathology caused by misfolded α-syn: (i) reducing its aggregation, (ii) controlling its propagation through the brain, (iii) increasing the clearance rate of α-syn, and (iv) stabilizing existing misfolding α-syn so the neurodegeneration does not worsen [4].

A comprehensive understanding of the aggregation process of α-syn could be achieved by examining the variability in its amino acid sequence. Certain mutations, namely A30P, E46K, H50Q, G51D, and A53T, have been identified as inducers of early onset PD, with A53T and A30P mutations specifically accelerating oligomerization [18,20]. Additionally, various post-translational modifications (PTMs), including acetylation, oxidation, nitration, and ubiquitination, have been observed in the Lewy bodies of PD patients. These PTMs may impact the aggregation and toxicity of α-syn [22]. Among these PTMs, phosphorylation takes place at S129 and S87, as well as Y125, Y133, and Y136 *in vivo*, playing a crucial role in oligomerization, Lewy body formation, and neurotoxicity [22]. Furthermore, it is suggested that PTMs may disrupt the mitochondrial function of α-syn by impeding protein import [18].

Treatments for synucleinopathies are currently limited. However, there is evidence that polyphenols are potentially effective at inhibiting α-syn aggregation [17]. These compounds are suggested to protect against oligomerization and fibril formation, theoretically preventing the accumulation of synucleinopathy-causing aggregates. Unfortunately, it is doubtful that polyphenols can cross the blood-brain barrier due to their chemical properties [17]. There is also evidence that the combination of therapies can effectively reduce the accumulation and propagation of α-syn [23]. Combination therapy allows patients to receive more than one therapeutic agent for their specific disease. More specifically, the combination of curcumin and piperine, two antioxidant supplements, has successfully protected against α-syn_agg_-driven PS [24]. This therapeutic approach will need to be studied further regarding synucleinopathies; however, it has been found to be successful against cancer and autoimmune diseases [23].

There are millions who suffer from PD, and its disruptions in motor behavior are oftentimes life-altering for those affected [25]. To gain a better understanding of α-syn's aggregation propensity, this study looks for similarities between various animal species to human α-syn. Identifying common fragments with a higher propensity for self-aggregation may lead to further insight into their use in drug screening to find potential treatment of synucleinopathies. The comparison of aggregation across species in comparison to humans may allow for the identification of further α-syn aggregation-inhibiting compounds as well as a non-rodent animal model for future studies of synucleinopathies.

## 2 Materials and methods

### 2.1 Chemical and α-syn peptides

Thioflavin-T (ThT) was purchased from Alfa Aesar (Ward Hill, MA). Recombinant human α-syn (140 amino acids) was obtained from rPeptide (Watkinsville, GA). All the human α-syn fragment peptides of 25 amino acids were prepared synthetically by GenScript (Piscataway, New Jersey) and present purity levels ≥95 %. All the species α-syn fragment peptides of 25 amino acids were prepared synthetically by GenScript (Piscataway, New Jersey) and present purity levels ranging between 50 % and 70 %. Of note, all amino acid sequences were verified through their respective NCBI protein IDs: human α-syn (NP_001362215), Abingdon Island giant tortoise (XP_032637993), Amur tiger (XP_042840122), common wall lizard (XP_028600608), Greenland sleeper shark (AUF74481), turkey (XP_019470104), and western terrestrial garter snake (XP_032080224).

### 2.2 Thioflavin T fluorescence (ThT) assays

A ThT dye fluorescence assay was performed using α-syn peptide fragments from all species in our bank to evaluate the kinetics of aggregation. Each α-syn peptide fragment (GenScript, Piscataway, NJ) was dissolved in 20 mM Tris-HCl (pH 7.4), resulting in a stock solution of 1 mM. ThT assays were performed using each peptide at a final concentration of 100 μM and as described in Ref. [26]. Buffer consisted of 10 mM PBS (pH 7.4) with 0.5 mM SDS and 300 mM of NaCl. Histograms were done using the mean of the maximum fluorescence intensity at the end of the kinetics and the standard error of the mean (SEM) with the GraphPad Prism version 8.1.1.

### 2.3 Transmission electron microscopy (TEM)

A transmission electron microscopy (TEM) analysis was performed using 10 μL of each α-syn fragment at a concentration of 100 μM (pre-incubated for one week in 10 mM PBS with 0.5 mM SDS and 300 mM of NaCl at 37 °C), applied to a 400-mesh Formvar-carbon-coated copper grid (Electron Microscopy Sciences, Hatfield, PA). The grids were incubated for 1 min, washed three times with distilled water, air-dried, then incubated for another minute in fresh 1 % uranyl acetate, and air-dried once more. Fibril visualization was achieved using transmission electron microscopy (JEOL 1400 Flash, Japan) at a voltage of 100 kV and magnification of 40k, following the completion of each kinetics of aggregation.

## 3 Results

### 3.1 In vitro aggregation propensity assessment

Fibril formation for α-syn peptides was determined using ThT fluorescence assays and TEM imaging. While ThT is a highly sensitive test, it does not specifically identify amyloid-like fibrils and cannot detect early aggregations such as oligomers and protofibrils [27]. Therefore, confirming ThT results with other biophysical methods like TEM is crucial. TEM is highly accurate and can diagnose AA amyloids alone, proving its reliability in identifying fibril formation [[28], [29], [30], [31]]. Circular dichroism (CD) results are provided to address any inconsistencies between the ThT and TEM results (Figs. S5–S8).

Additionally, α-syn fragments 1–25, 37–61, and 62–86 were selected for further study with various species. α-Syn 1–25 was chosen as a negative control, α-syn 37–61 was selected due to the presence of the A53T mutation in several species, and α-syn 62–86 was chosen for its high aggregation propensity and location in the NAC region.

### 3.2 Thioflavin T fluorescence assay

**Fig. 1:**
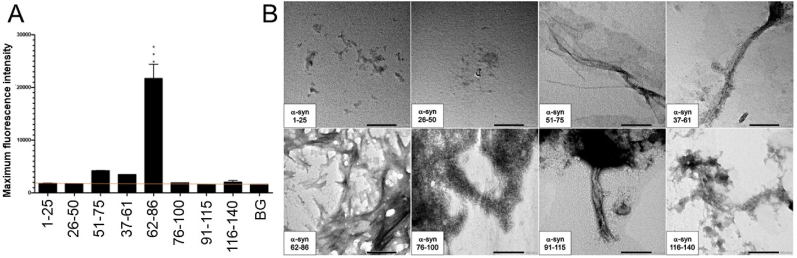
Formation of amyloid-like fibrils using the human α-syn fragments 51–75, 37–61, 62–86, 76–100, and 116-140 by ThT. The human α-syn fragments 1–25 and 26–50 did not generate fibrils by TEM. A) Histogram of Thioflavin T results for all human α-syn peptide fragments, including the background (BG) indicated by an orange line. A one-way ANOVA with Dunnett's multiple comparison test was conducted. The bars represent the mean fluorescence, with error bars showing the SEM for three replicates. Statistical significance is indicated as ***p < 0.001. B) Transmission electron microscopy (TEM) imaging for all human α-syn peptide fragments incubated at 37 °C for 72h at a magnification of 40k. Scale bar = 200 nm.

**Fig. 2:**
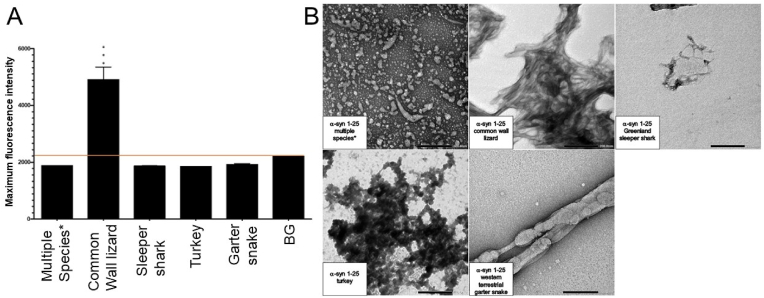
Formation of amyloid-like fibrils for only the common wall lizard of the α-syn 1–25 peptide fragment by ThT. The α-syn 1–25 peptide fragments from the common wall lizard, Greenland sleeper shark, and turkey generated fibrils as shown in TEM images. A) Histogram of Thioflavin T results for all examined species of the 1–25 α-syn peptide fragment, including the background (BG) indicated by an orange line. A one-way ANOVA with Dunnett's multiple comparison test was conducted. The bars represent the mean fluorescence, with error bars showing the SEM for three replicates. Statistical significance is indicated as ***p < 0.001. Multiple species*, including the human, Abingdon Island giant tortoise, and Amur tiger, share a common amino acid sequence. B) Transmission electron microscopy imaging for all examined species of the α-syn 1–25 peptide fragment incubated at 37 °C for 72h at a magnification of 40k. Multiple species*, including the human, Abingdon Island giant tortoise, and Amur tiger, share a common amino acid sequence. Scale bar = 200 nm. (For interpretation of the references to colour in this figure legend, the reader is referred to the Web version of this article.)

The kinetics of fibril formation for each fragment peptides were monitored with the ThT binding assays. The maximum fluorescence intensity obtained at the end of the kinetics. Histograms (Fig. 1, Fig. 2) present the average values with SEM for each fragment. Human α-syn fragments 1–25, 26–50, and 37–61 represent the N-terminal region. α-Syn 51–75 is part of both the N-terminal and non-amyloid-β component (NAC) regions. α-Syn 62–86 belongs to the NAC region. α-Syn fragments 76–100 and 91–115 cover the NAC and C-terminal regions. α-Syn 116–140 belongs to the C-terminal region. Notably, α-syn fragments 1–25, 26–50, and 91–115 did not form fibrils on ThT assays, while α-syn fragments 51–75, 37–61, 62–86, 76–100, and 116–140 exhibited higher fluorescence intensity (i.e. indicative of fibril formation) (Fig. 1A) and generated typical sigmoidal ThT curves as shown in Fig. S1. The elevated fluorescence intensity was not statistically significant for the α-syn fragments 51–75, 37–61, 76–100 and 116–140. However, the sigmoidal curves obtained from these α-syn fragments are indicative of fibril formation (Fig. S1).

Only one examined species of the α-syn 1–25 fragment, the common wall lizard (*Podarcis muralis*), was shown to form fibrils on ThT. The remaining species, including the human (*Homo sapiens*), Abingdon Island giant tortoise (*Chelonoidis abingdonii)*, Amur tiger (*Panthera tigris altaica*), Greenland sleeper shark (*Somniosus microcephalus*), turkey (*Meleagris gallopavo*), and western terrestrial garter snake (*Thamnophis elegans)* did not exhibit fibril formation on ThT (Fig. 2A) and resulted in ThT flat fluorescence signal during the kinetics (Fig. S2).

**Fig. 3:**
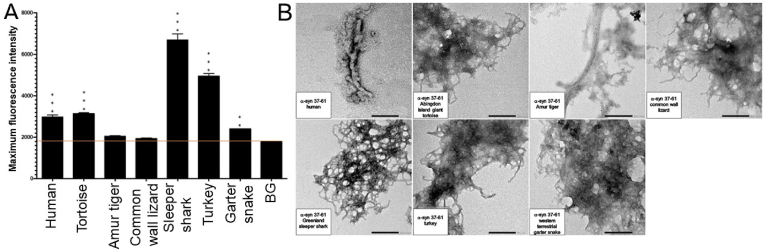
Formation of amyloid-like fibrils for all species of the α-syn 37–61 peptide fragment by ThT and TEM. A) Histogram of Thioflavin T results for all examined species of the 37–61 α-syn peptide fragment, including the background (BG) indicated by an orange line. A one-way ANOVA with Dunnett's multiple comparison test was conducted. The bars represent the mean fluorescence, with error bars showing the SEM for three replicates. Statistical significance is indicated as **p < 0.01 and ***p < 0.001. B) Transmission electron microscopy imaging for all examined species of the α-syn 37–61 peptide fragment incubated at 37 °C for 72h at a magnification of 40k. Scale bar = 200 nm. TEM images of human fragment 37–61 is published in Ref. [26]. (For interpretation of the references to colour in this figure legend, the reader is referred to the Web version of this article.)

All examined species of the α-syn 37–61 fragment, including the human (*Homo sapiens*), Abingdon Island giant tortoise (*Chelonoidis abingdonii)*, Amur tiger (*Panthera tigris altaica*), common wall lizard (*Podarcis muralis*), Greenland sleeper shark (*Somniosus microcephalus*), turkey (*Meleagris gallopavo*), and western terrestrial garter snake (*Thamnophis elegans)* were shown to form fibrils on ThT (Fig. 3A). These fragments resulted in elvelated ThT kinetic curves when compared with the background signal (Fig. S3).

**Fig. 4:**
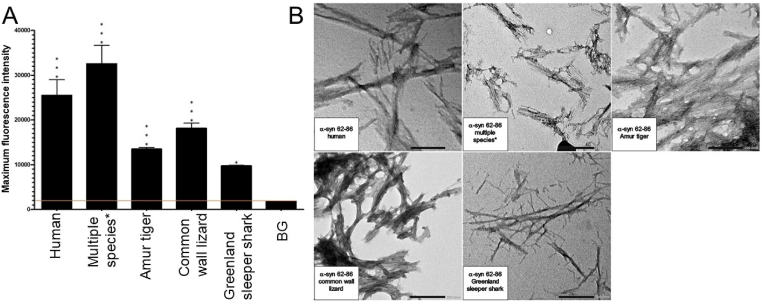
Formation of amyloid-like fibrils for all species of the α-syn 62–86 peptide fragment by ThT and TEM. A) Histogram of Thioflavin T results for all examined species of the 62–86 α-syn peptide fragment, including the background (BG) indicated by an orange line. A one-way ANOVA with Dunnett's multiple comparison test was conducted. The bars represent the mean fluorescence, with error bars showing the SEM for three replicates. Statistical significance is indicated as * p < 0.05 and ***p < 0.001. Multiple species*, including the Abingdon Island giant tortoise, turkey, and western terrestrial garter snake, share a common amino acid sequence. B) Transmission electron microscopy imaging for all examined species of the α-syn 62–86 peptide fragment incubated at 37 °C for 72h at a magnification of 40k. Multiple species*, including the Abingdon Island giant tortoise, turkey, and western terrestrial garter snake, share a common amino acid sequence. Scale bar = 200 nm. TEM images of human fragment 62–86 is published in Ref. [26]. (For interpretation of the references to colour in this figure legend, the reader is referred to the Web version of this article.)

All examined species of the α-syn 62–86 fragment, including the human (*Homo sapiens*), Abingdon Island giant tortoise (*Chelonoidis abingdonii)*, Amur tiger (*Panthera tigris altaica*), common wall lizard (*Podarcis muralis*), Greenland sleeper shark (*Somniosus microcephalus*), turkey (*Meleagris gallopavo*), and western terrestrial garter snake (*Thamnophis elegans)* were shown to form fibrils according to the ThT assays (Fig. 4A). The kinetics of fibril formation detected by ThT of the α-syn 62–86 fragments are presented in Fig. S4.

**Fig. 5:**
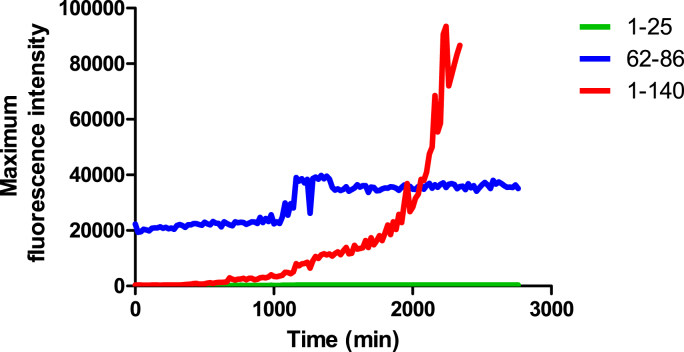
Formation of amyloid-like fibrils for the human α-syn 62–86 peptide fragment. Comparison of Thioflavin T fibrilization kinetics for the α-syn 1–25 peptide fragment and α-syn 62–86 peptide fragment, relative to the full-length (1–140) α-syn protein. All samples were tested at a concentration of 100 μM. Overflow is seen at the full-length α-syn protein.

Additionally, an image is included showing the ThT curve of the human α-syn 1–25 and α-syn 62–86 fragments compared to the full-length alpha-synuclein protein. The α-syn 62–86 fragment exhibited fibril formation on ThT, while the α-syn 1–25 fragment did not show any substantial elevated fluorescence intensity, indicative of no fibril formation. The full-length peptide at 100 μM resulted in overflow (Fig. 5).

### 3.3 Transmission electron microscopy fibrillation patterns

The direct visualization of incubated samples by transmission electron microscopy (TEM) for the human fragments indicated that α-syn fragments 1–25 and 26–50 did not form fibrils. In contrast, α-syn fragments 51–75, 37–61, 62–86, 76–100, 91–115, and 116–140 showed mature fibrils by TEM (Fig. 1B).

The TEM assessment of the α-syn 1–25 fragments confirmed that the common wall lizard (*Podarcis muralis*), Greenland sleeper shark (*Somniosus microcephalus*), and turkey (*Meleagris gallopavo*) validated the formation of amyloid-like fibrils (Fig. 2). Conversely, the human (*Homo sapiens*), Abingdon Island giant tortoise (*Chelonoidis abingdonii)*, Amur tiger (*Panthera tigris altaica*), and western terrestrial garter snake (*Thamnophis elegans)* did not form fibrils (Fig. 2B).

The TEM analyses of the α-syn 37–61 fragments indicated that the human (*Homo sapiens*), Abingdon Island giant tortoise (*Chelonoidis abingdonii)*, Amur tiger (*Panthera tigris altaica*), common wall lizard (*Podarcis muralis*), Greenland sleeper shark (*Somniosus microcephalus*), turkey (*Meleagris gallopavo*), and western terrestrial garter snake (*Thamnophis elegans)* were capable to form fibrils (Fig. 3B).

The TEM for the species of the α-syn 62–86 fragment confirmed that the human (*Homo sapiens*), Abingdon Island giant tortoise (*Chelonoidis abingdonii)*, Amur tiger (*Panthera tigris altaica*), common wall lizard (*Podarcis muralis*), Greenland sleeper shark (*Somniosus microcephalus*), turkey (*Meleagris gallopavo*), and western terrestrial garter snake (*Thamnophis elegans)* generated mature fibrils (Fig. 4B).

### 3.4 Amyloidogenicity of peptide fragments as determined by ThT assay and TEM

The aggregation propensity of the human peptide fragments varied greatly. Human α-syn fragments 1–25 and 26–50 demonstrated to not form fibrils through their negative TEM and ThT results. However, human α-syn fragments 51–75, 37–61, 62–86, 76–100, and 116–140 displayed fibril formation in their positive TEM and ThT results. Human α-syn fragment 91–115 yielded inconclusive results, with a negative ThT result but a positive TEM result. However, given its positive CD result, fragment 91–115 is classified as likely positive (Fig. S5).

The α-syn 1–25 peptide fragments displayed a low level of aggregation propensity. Only one fragment, specifically the common wall lizard (*Podarcis muralis*), exhibited fibril formation through its positive ThT and TEM results. The human (*Homo sapiens*), Abingdon Island giant tortoise (*Chelonoidis abingdonii)*, and western terrestrial garter snake (*Thamnophis elegans)* did not form fibrils in their negative ThT and TEM results. The Greenland sleeper shark (*Somniosus microcephalus*) and turkey (*Meleagris gallopavo*) were inconclusive with a positive TEM result and a negative ThT result (Table 2). However, given their positive CD results, α-syn 1–25 fragments Greenland sleeper shark and turkey are classified as likely positive (Fig. S6).

The α-syn 37–61 peptide fragments displayed a high level of aggregation propensity. All fragments provided positive ThT and TEM results. The α-syn 37–61 fragments that formed fibrils included the human (*Homo sapiens*), Abingdon Island giant tortoise (*Chelonoidis abingdonii)*, Amur tiger (*Panthera tigris altaica*), common wall lizard (*Podarcis muralis*), Greenland sleeper shark (*Somniosus microcephalus*), turkey (*Meleagris gallopavo*), and western terrestrial garter snake (*Thamnophis elegans)* (Table 2).

The α-syn 62–86 peptide fragments displayed a very high level of aggregation propensity. All fragments provided positive ThT and TEM results. The α-syn 62–86 fragments that formed fibrils included the human (*Homo sapiens*), Abingdon Island giant tortoise (*Chelonoidis abingdonii)*, Amur tiger (*Panthera tigris altaica*), common wall lizard (*Podarcis muralis*), Greenland sleeper shark (*Somniosus microcephalus*), turkey (*Meleagris gallopavo*), and western terrestrial garter snake (*Thamnophis elegans)* (Table 2).

## 4 Discussion

The combined data strongly indicate that the α-syn fragments derived from the amino acid 37–61 and 62–86 regions exhibit a high susceptibility to aggregation (please see results summarized in Table 1, Table 2). Fibril formation was observed across all species for these regions. In contrast, α-syn regions 1–25 and 26–50, which correspond to the N-terminal region, showed a lower tendency for aggregation compared to regions 51–75, 37–61, 62–86, 76–100, and 116–140, which include the NAC and C-terminal regions, with the exception of fragment 37–61. Notably, the α-syn 91–115 region exhibited probable aggregation, evidenced by fibril formation on TEM and CD assays, but minimal fluorescence on ThT. Additionally, the α-syn 1–25 fragment showed very poor amyloidogenicity in most species, except for the common wall lizard, which showed positive results in both TEM and ThT, and the Greenland sleeper shark and turkey, which were positive on TEM and CD but negative on ThT. The α-syn 37–61 fragments of the western terrestrial garter snake, turkey, Abingdon Island giant tortoise, common wall lizard, and Amur tiger all possess the A53T mutation, which might be linked with early-onset PD (Table 5). Of these fragments, the common wall lizard and western terrestrial garter snake displayed a higher propensity for aggregation than the human counterpart, as demonstrated by ThT analysis (Fig. 1A). These fragments may serve as positive controls in future studies, though their direct clinical relevance is yet to be established.

**Table 1:** Summary of the fibrillar aggregation properties for human α-syn peptide fragment, ThT, Thioflavin T; TEM, transmission electron microscopy.

Peptide Fragment	ThT	TEM	CD	Amyloidogenicity
α-syn 1-25	–	–	–	–
α-syn 26-50	–	–	–	–
α-syn 51-75	+	+	+	+
α-syn 37-61	+	+	+	+
α-syn 62-86	+	+	+	+
α-syn 76-100	+	+	+	+
α-syn 91-115	–	+	+	Likely +
α-syn 116-140	+	+	+	+

**Table 2:** Summary of the fibrillar aggregation properties of the examined species of the α-syn fragments 1–25, 37–61, and 62–86, ThT, Thioflavin T; TEM, transmission electron microscopy.

Fragment 1–25 Species	ThT	TEM	CD	Amyloidogenicity
Human *(Homo sapiens)*	–	–	–	–
Abingdon Island Giant Tortoise *(Chelonoidis abingdonii)*	–	–	–	–
Amur Tiger *(Panthera tigris altaica)*	–	–	–	–
Common Wall Lizard *(Podarcis muralis)*	+	+	–	+
Greenland Sleeper Shark *(Somniosus microcephalus)*	–	+	+	Likely +
Turkey *(Meleagris gallopavo)*	–	+	+	Likely +
Western Terrestrial Garter Snake *(Thamnophis elegans)*	–	–	+	Likely -

**Fragment 37–61 Species**	**ThT**	**TEM**	**CD**	**Amyloidogenicity**
Human *(Homo sapiens)*	+	+	+	+
Abingdon Island Giant Tortoise *(Chelonoidis abingdonii)*	+	+	+	+
Amur Tiger *(Panthera tigris altaica)*	+	+	+	+
Common Wall Lizard *(Podarcis muralis)*	+	+	+	+
Greenland Sleeper Shark *(Somniosus microcephalus)*	+	+	+	+
Turkey *(Meleagris gallopavo)*	+	+	+	+
Western Terrestrial Garter Snake *(Thamnophis elegans)*	+	+	+	+

**Fragment 62–86 Species**	**ThT**	**TEM**	**CD**	**Amyloidogenicity**
Human *(Homo sapiens)*	+	+	+	+
Abingdon Island Giant Tortoise *(Chelonoidis abingdonii)*	+	+	+	+
Amur Tiger *(Panthera tigris altaica)*	+	+	+	+
Common Wall Lizard *(Podarcis muralis)*	+	+	+	+
Greenland Sleeper Shark *(Somniosus microcephalus)*	+	+	+	+
Turkey *(Meleagris gallopavo)*	+	+	+	+
Western Terrestrial Garter Snake *(Thamnophis elegans)*	+	+	+	+

Expanding on these findings, it becomes increasingly evident that the NAC region plays an important role in α-syn misfolding and fibrillogenesis. Fragments containing any portion of the NAC region, specifically α-syn fragments 51–75, 62–86, 76–100, and 91–115 displayed fibril formation in the transmission electron microscopy (TEM) results. Additionally, all these NAC-containing fragments, except for α-syn 91–115, exhibit fibril formation in the Thioflavin T (ThT) assay results. Notably, the α-syn 62–86 fragment, which is composed entirely of the NAC region, has the highest level of aggregation among all the fragments. Further examination of this fragment across different species revealed significant fibril formation, supporting the idea that the NAC region may directly influence the aggregation propensity of alpha-synuclein. It is worth noting that α-syn 91–115, with the lowest NAC content (20 %), showed minimal fibril formation. Looking at the study by Rodriguez et al., they found that the NAC region, specifically the NACore region (residues 68–78), plays a significant role in the misfolding of α-syn and related cytotoxicity, suggesting that the structure of the NACore resembles the toxic fibrils found in α-syn associated with PD [32]. Therefore, our consistent positive results with the α-syn 62–86 fragment, which contains the entire NACore region, may suggest a significant correlation between the misfolding of α-syn and the presence of the NAC region. Studying the NAC region is critical to understanding the impact of α-syn in neurodegeneration.

Furthermore, our results demonstrate the consistent positive amyloidogenicity exhibited by the peptide fragments of the α-syn 37–61 region. Notably, several fragments, namely the Abingdon Island giant tortoise, Amur tiger, common wall lizard, turkey, and western terrestrial garter snake all contain a shared point mutation, A53T (Table 5). The A53T mutation has been identified as a key factor impairing lipid binding while enhancing aggregation properties, rendering it the fastest-aggregating among the point mutations linked to α-syn [20,25]. Additionally, a study by Gallardo et al. highlighted the A53T mutation's capability to enhance α-syn nucleation and elongation [33]. These findings reinforce that there may be a correlation between fibril-forming capacity and the presence of the A53T mutation.

The variations in amino acid composition among various fragments have revealed interesting results (see Table 3, Table 4, Table 5, Table 6). The propensity of a fragment to aggregate appears to be potentially influenced by its acidic amino acid content; fewer acidic residues often correlate with increased aggregation and fibril formation. For example, the α-syn 62–86 fragment, with just one acidic amino acid, exhibits the highest susceptibility to aggregate of the fragments. In contrast, α-syn fragments 91–115 and 116–140, which have the highest numbers of acidic amino acids, (six and nine, respectively), demonstrate lower aggregation tendencies (Table S1). Moreover, fragments rich in valine, a nonpolar amino acid, may show higher aggregation potential. Both α-syn fragments 51–75 and 62–86 have valine as the most frequent amino acid, appearing seven times in each sequence. Both fragments exhibited fibril formation in both ThT and TEM analyses, resulting in the two highest aggregation propensities. Conversely, α-syn fragments 91–115 and 116–140, with minimal valine presence, display lower aggregation. Thus, the abundance of valine and the presence of acidic residues may serve as indicators of a fragment's aggregation potential. Future studies should be conducted to experimentally determine if the amino acid composition plays any relevance in aggregation propensity.

**Table 3:** Amino acid sequences of each human α-syn fragment peptide utilized in this study. These fragments span the entire 140 amino acid region of α-syn, including the N-terminal, NAC region, and C-terminal.

**Peptide Fragment**	**1**	**2**	**3**	**4**	**5**	**6**	**7**	**8**	**9**	**10**	**11**	**12**	**13**	**14**	**15**	**16**	**17**	**18**	**19**	**20**	**21**	**22**	**23**	**24**	**25**
**α-syn1-25**	**M**	**D**	**V**	**F**	**M**	**K**	**G**	**L**	**S**	**K**	**A**	**K**	**E**	**G**	**V**	**V**	**A**	**A**	**A**	**E**	**K**	**T**	**K**	**Q**	**G**
Peptide Fragment	26	27	28	29	30	31	32	33	34	35	36	37	38	39	40	41	42	43	44	45	46	47	48	49	50
**α-syn26**–**50**	**V**	**A**	**E**	**A**	**A**	**G**	**K**	**T**	**K**	**E**	**G**	**V**	**L**	**Y**	**V**	**G**	**S**	**K**	**T**	**K**	**E**	**G**	**V**	**V**	**H**
Peptide Fragment	51	52	53	54	55	56	57	58	59	60	61	62	63	64	65	66	67	68	69	70	71	72	73	74	75
**α-syn51**–**75**	**G**	**V**	**A**	**T**	**V**	**A**	**E**	**K**	**T**	**K**	**E**	**Q**	**V**	**T**	**N**	**V**	**G**	**G**	**A**	**V**	**V**	**T**	**G**	**V**	**T**
Peptide Fragment	37	38	39	40	41	42	43	44	45	46	47	48	49	50	51	52	53	54	55	56	57	58	59	60	61
**α-syn37**–**61**	**V**	**L**	**Y**	**V**	**G**	**S**	**K**	**T**	**K**	**E**	**G**	**V**	**V**	**H**	**G**	**V**	**A**	**T**	**V**	**A**	**E**	**K**	**T**	**K**	**E**
Peptide Fragment	62	63	64	65	66	67	68	69	70	71	72	73	74	75	76	77	78	79	80	81	82	83	84	85	86
**α-syn62**–**86**	**Q**	**V**	**T**	**N**	**V**	**G**	**G**	**A**	**V**	**V**	**T**	**G**	**V**	**T**	**A**	**V**	**A**	**Q**	**K**	**T**	**V**	**E**	**G**	**A**	**G**
Peptide Fragment	76	77	78	79	80	81	82	83	84	85	86	87	88	89	90	91	92	93	94	95	96	97	98	99	100
**α-syn76**–**100**	**A**	**V**	**A**	**Q**	**K**	**T**	**V**	**E**	**G**	**A**	**G**	**S**	**I**	**A**	**A**	**A**	**T**	**G**	**F**	**V**	**K**	**K**	**D**	**Q**	**L**
Peptide Fragment	91	92	93	94	95	96	97	98	99	100	101	102	103	104	105	106	107	108	109	110	111	112	113	114	115
**α-syn91**–**115**	**A**	**T**	**G**	**F**	**V**	**K**	**K**	**D**	**Q**	**L**	**G**	**K**	**N**	**E**	**E**	**G**	**A**	**P**	**Q**	**E**	**G**	**I**	**L**	**E**	**D**
Peptide Fragment	116	117	118	119	120	121	122	123	124	125	126	127	128	129	130	131	132	133	134	135	136	137	138	139	140
**α-syn 116**–**140**	**M**	**P**	**V**	**D**	**P**	**D**	**N**	**E**	**A**	**Y**	**E**	**M**	**P**	**S**	**E**	**E**	**G**	**Y**	**Q**	**D**	**Y**	**E**	**P**	**E**	**A**

**Table 4:**
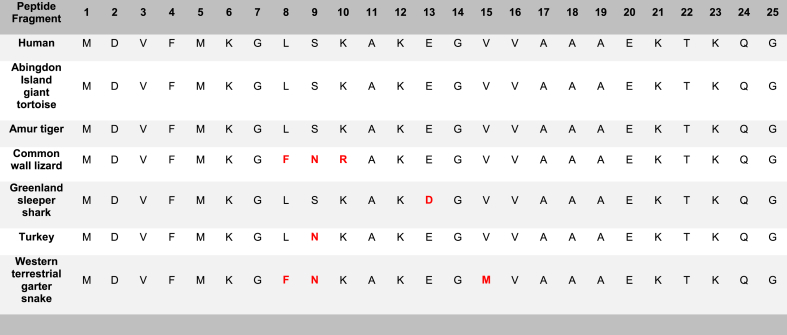
**Amino acid sequences of the α-syn1–25 series. This1–25fragment is representative of the N-terminal amphipathic (1-60) region of α-syn.** Amino acid variations are highlighted in red.

**Table 5:**
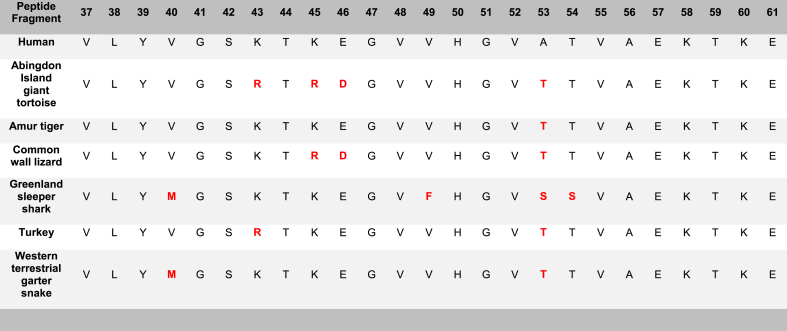
**Amino acid sequences of the α-syn37–61 series. This37**–**61fragment is representative of the N-terminal amphipathic (1-60) region ofα-syn.** Amino acid variations are highlighted in red.

**Table 6:**
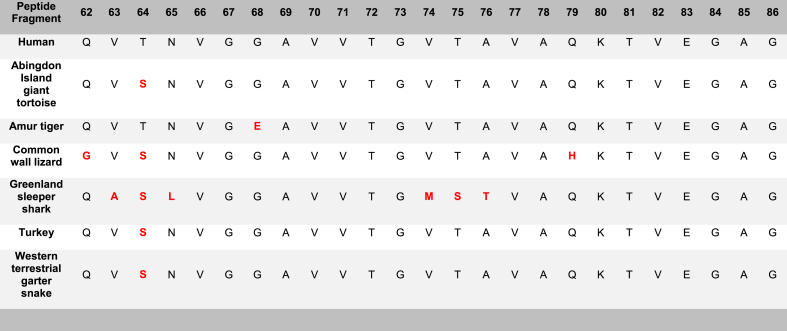
**Amino acid sequences of the α-syn62–86 series. This62**–**86fragment is representative of the hydrophobic non-amyloid-β component (NAC) (61–95) region of α-syn.** Amino acid variations are highlighted in red.

Limitations of this study stemmed from the inability to perfectly replicate exact physiologic conditions, so our results are not a perfect indicator of how these fragments would or would not misfold *in vivo*. ThT assays vary depending on sample concentration and incubation time, amongst other factors. Further investigation may lead to finding potential animal models for the study of PD and other neurodegenerative diseases. Comparing similarities (such as life span) between species that are prone to misfolded α-syn and humans may lead to further insight into how and why α-syn causes disease. Future directions of this work would be cell culture to determine the cytotoxicity of specific fragments, and additional repeats of biophysical assays for further confirmation of our results.

## 5 Conclusion

In this study, we compiled several α-syn peptide fragments (25 amino acids in length) to investigate the misfolding of α-syn. Our findings revealed that the human α-syn fragments 51–75, 37–61, 62–86, 76–100, and 116–140 exhibited a higher tendency to aggregate when compared to the human α-syn fragments 1–25, 26–50, and 91–115. These results indicate a potential association between the presence of the NAC region and the A53T mutation, both of which may influence fibril formation in α-syn. Additionally, the high prevalence of valine and low quantity of acidic amino acids may play a role in influencing the aggregation potential of α-syn. We utilized Thioflavin T fluorescence assay and transmission electron microscopy to visualize fibril formation and monitor the kinetics of aggregation. These findings enhance the understanding of α-syn aggregation and the molecular processes driving neurodegenerative diseases, including Parkinson's disease. Future research can provide an in-depth experimental investigation into the effects of the A53T mutation, NAC region, and specific amino acids on aggregation propensity.

## Funding

The authors are grateful for the ASIP Summer Research Opportunity Program in Pathology (SROPP).

## CRediT authorship contribution statement

**Natalie G. Horgan:** Writing – review & editing, Writing – original draft, Validation, Investigation, Formal analysis, Data curation. **Annie M. McCarty:** Writing – review & editing, Writing – original draft, Investigation, Formal analysis. **Ashley A. Hetak:** Writing – original draft, Investigation, Formal analysis, Conceptualization. **Hailey B. Penticoff:** Writing – original draft, Formal analysis, Data curation. **Jessica S. Fortin:** Writing – review & editing, Writing – original draft, Validation, Supervision, Investigation, Funding acquisition, Formal analysis, Data curation, Conceptualization.

## Declaration of competing interest

The authors declare that they have no known competing financial interests or personal relationships that could have appeared to influence the work reported in this paper.

## Data Availability

The data are presented in the article.
