# Supplementary material for: Understanding alpha-synuclein aggregation propensity in animals and humans
Source: Biochem Biophys Rep. 2024 Aug 14;39:101810. doi: 10.1016/j.bbrep.2024.101810 (PMC11367636; doi:10.1016/j.bbrep.2024.101810)

**Understanding alpha-synuclein aggregation propensity in animals and humans**

Natalie G. Horgan^1^, Annie M. McCarty^1^, Ashley A. Hetak^2^, Hailey B. Penticoff^2^, and Jessica S. Fortin^1*^

^1^Department of Basic Medical Sciences, College of Veterinary Medicine, Purdue University, West Lafayette, IN 47906.

^2^Department of Pathobiology and Diagnostic Investigation, College of Veterinary Medicine, Michigan State University, East Lansing, MI 48824.

Contact Jessica S. Fortin [fortinj@purdue.edu](mailto:fortinj@purdue.edu) Department of Basic Medical Sciences, College of Veterinary Medicine, Purdue University, 610 Purdue Mall, West Lafayette, IN 47907

**INDEX**

Diversity of amino acids…………………………………………………………………...Table S1

Kinetics of fibril formation (curves) by thioflavin T………………………………...Figures S1-S4

Analysis of secondary structures by circular dichroism (CD) ……………………….Figures S5-S8

Diversity of amino acids. The number of neutral nonpolar, neutral polar, acidic, and basic amino acids with the net charge for each region of the human α-syn are indicated in Table S1.

| **Peptide Fragment** | **α-syn region(s)** | **# Neutral, nonpolar** | **# Neutral, polar** | **# Acidic (-)** | **# Basic (+)** | **Most prominent amino acid**  **AA (#)- identification** | **Net charge** |
| --- | --- | --- | --- | --- | --- | --- | --- |
| α-syn 1-25 | N-terminal | 14 | 3 | 3 | 5 | K (5)- basic | 2 |
| α-syn 26-50 | N-terminal | 13 | 5 | 3 | 5 | V (5)- nonpolar | 2 |
| α-syn 51-75 | N-terminal & NAC | 14 | 7 | 2 | 2 | V (7)- nonpolar | 0 |
| α-syn 37-61 | NAC | 12 | 5 | 3 | 5 | V (6)- nonpolar | 2 |
| α-syn 62-86 | NAC | 16 | 7 | 1 | 1 | V (7)- nonpolar | 0 |
| α-syn 76-100 | C-terminal & NAC | 15 | 5 | 2 | 3 | A (6)- nonpolar | 1 |
| α-syn 91-115 | C-terminal & NAC | 12 | 4 | 6 | 3 | G (4)- nonpolar & E(4)- acidic | -3 |
| α-syn 116-140 | C-terminal | 10 | 6 | 9 | 0 | E (6)- acidic | -9 |
|  |  |  |  |  |  |  |  |

**Table S1. Summary of the diversity of amino acids across the synthetic fragments of the human α-synuclein, detailing the quantity of nonpolar, polar, acidic, and basic amino acids.**

**Kinetics of fibril formation as assessed by Thioflavin T (ThT) assays.** The curves representing the kinetics of fibril formation are shown in **Figures S1-S4** for each α-syn synthetic peptide examined in this study. Fragments were tested at 100 µM in 10 mM of PBS (pH 7.4) supplemented with 0.5 mM SDS and 300 mM NaCl. Thioflavin T was used at a concentration of 20 µM to track the formation of fibrils as indicated in the materials and methods section.

**Figure S1. Human α-syn fragments 51-75, 37-61, 62-86, 76-100, and 116-140 present an increase of thioflavin T (ThT) fluorescence intensity indicative of fibril formation.** Kinetics of human α-syn aggregation from regions 51-75, 37-61, 62-86, and 76-100 present a typical sigmoidal curve. Regions 1-25, 26-50, and 91-115 did not show an increase in ThT fluorescence intensity within the experimental conditions tested.


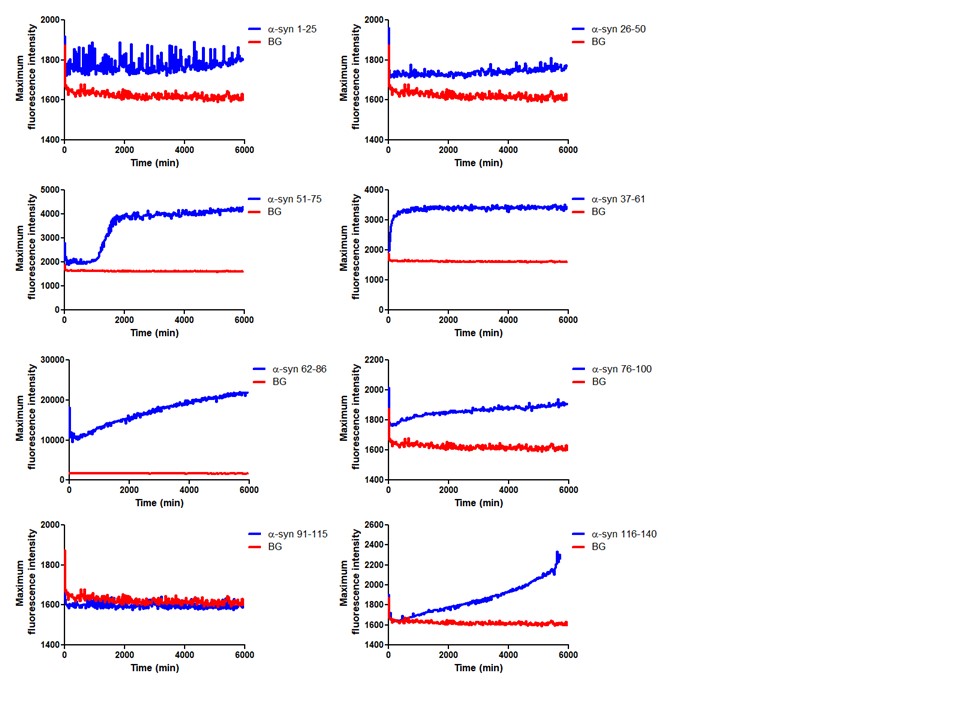


**Figure S2. Human, Abingdon Island giant tortoise, Amur tiger, Greenland sleeper shark, turkey, and western terrestrial garter snake α-syn 1-25 fragments do not demonstrate an increase of thioflavin T (ThT) fluorescence intensity indicative of no fibril formation within the experimental conditions tested.** Kinetics of the common wall lizard α-syn aggregation from regions 1-25 exhibits a typical sigmoidal curve. The asterisk indicates that the α-syn amino acid sequence region 1-25 is shared with the human, Abingdon Island giant tortoise, and Amur tiger.


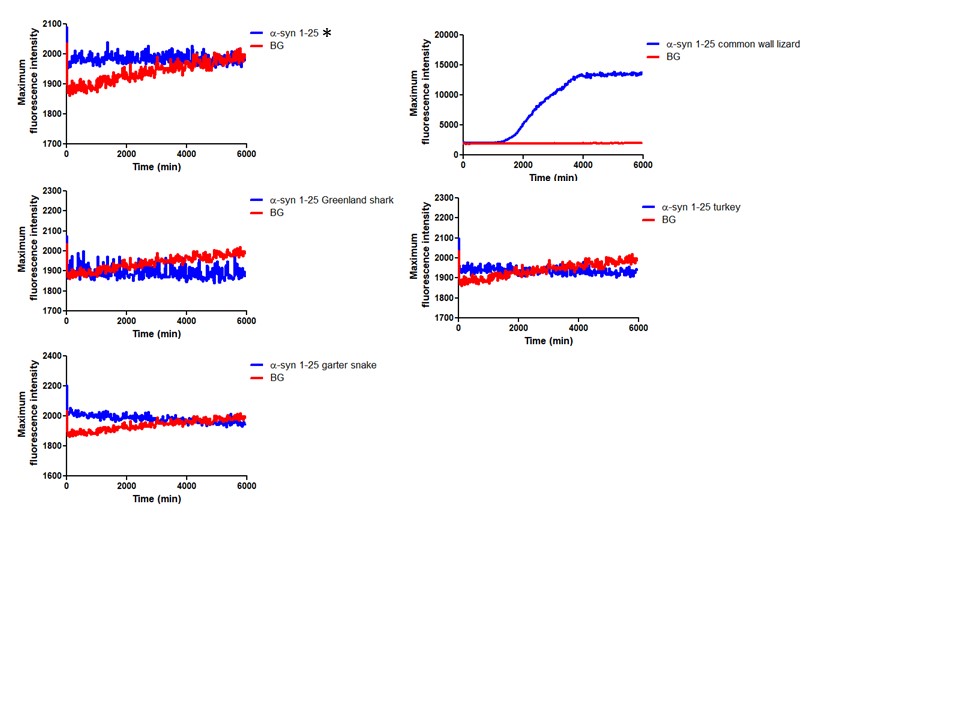


**Figure S3. Human, Abingdon Island giant tortoise, Amur tiger, common wall lizard, Greenland sleeper shark, turkey, and western terrestrial garter snake α-syn fragments 37-61 exhibit an increase of thioflavin T (ThT) fluorescence intensity indicative of fibril formation.**

**
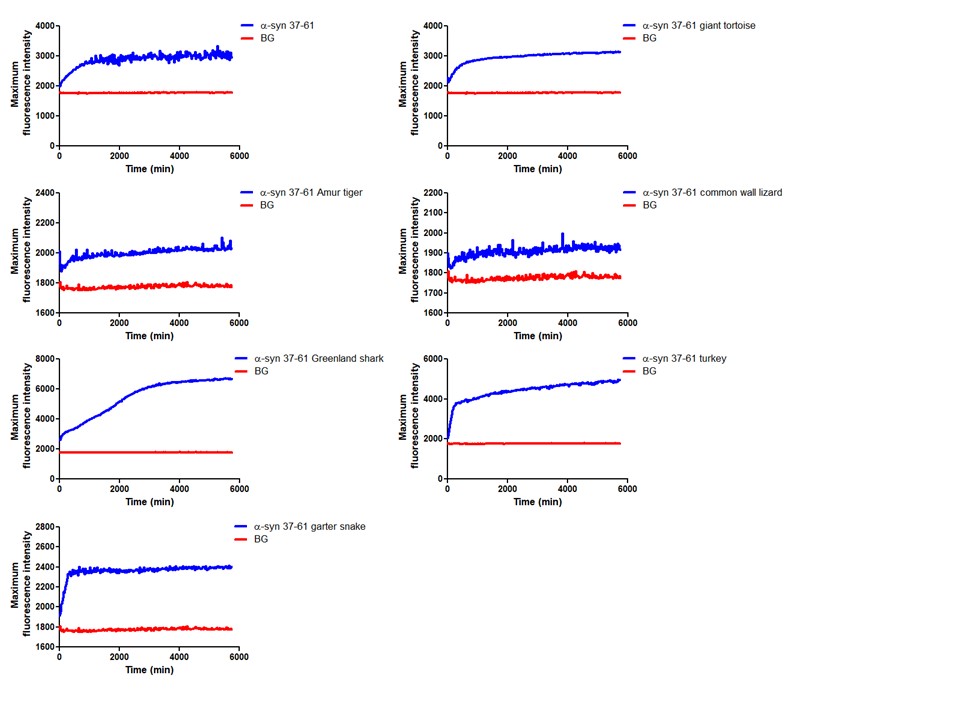
**

**Figure S4. Human, Abingdon Island giant tortoise, Amur tiger, common wall lizard, Greenland sleeper shark, turkey, and western terrestrial garter snake α-syn fragments 62-86 show typical sigmoidal-like curves based on the thioflavin T (ThT) assay, indicative of fibril formation.** The asterisk indicates that region 62-86 is shared with the Abingdon Island giant tortoise, turkey, and western terrestrial garter snake.


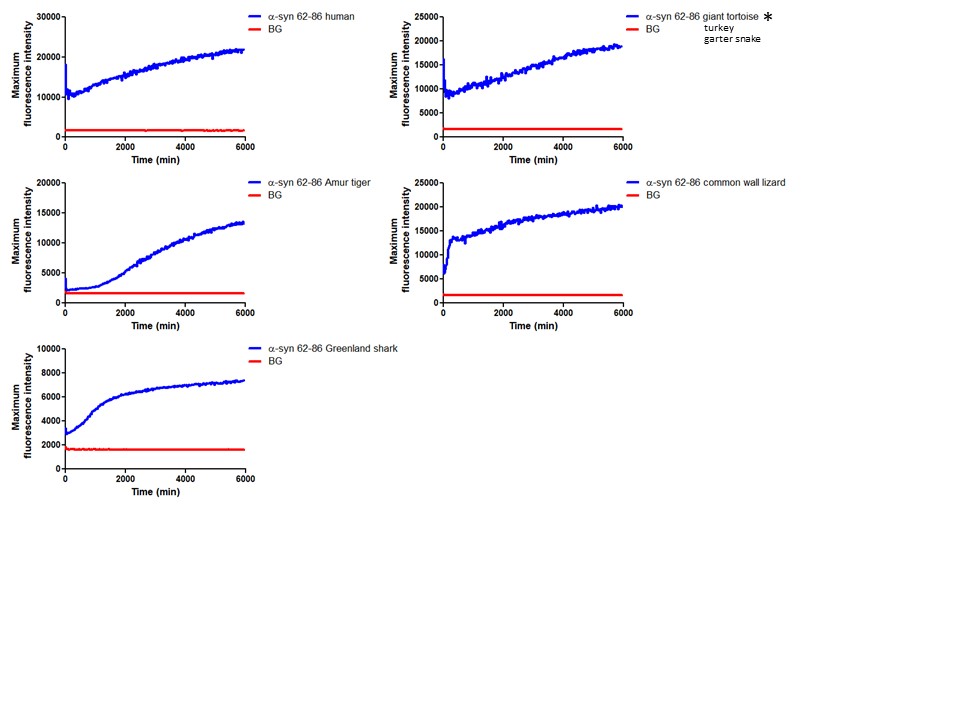


**Analysis of secondary structures by** **far-UV circular dichroism (CD).** To confirm ThT binding assays, CD spectra of the secondary structure of α-syn samples were recorded at 25°C under a constant flow of N_2_ using a JASCO-810 spectropolarimeter (Jasco, Easton, MD). Spectra were recorded over a wavelength range of 190–250 nm using a quartz cuvette of 1 mm path length and an instrument scanning speed of 100 nm/min, with a response time of 2 seconds and a bandwidth of 1 nm. All α-syn samples were dissolved to a final concentration of 100 µM in 10 mM PBS buffer (pH 7.4) containing 0.5 mM SDS and 300 mM of NaCl and incubated for 14 days at 37 ºC. Each result is given as the average scans taken of three measurements at room temperature. Recorded spectra are presented in **Figures S5-S8**.

**Figure S5. Human** **α-syn 51-75, 37-61, 62-86, 76-100, 91-115, 116-140 fragments exhibit a beta-plated sheet secondary structure as demonstrated by circular dichroism.** An alpha-helix with a beta-plated sheet is observed in regions 51-75, 37-61, 62-86, 91-115, and 116-140. Region 76-100 exhibits a beta-plated sheet secondary structure. Random coiled structures are observed in regions 1-25 and 26-50.


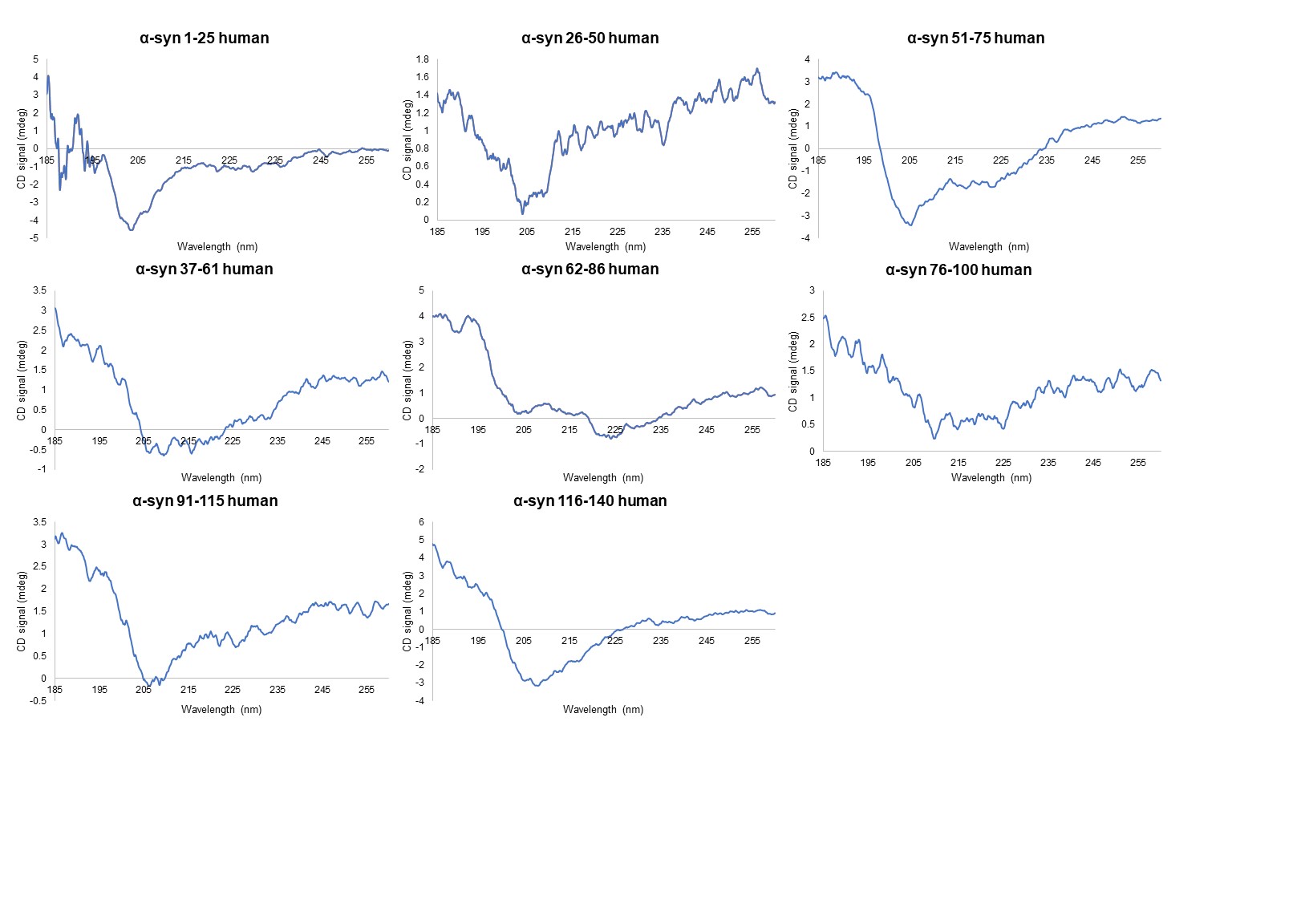


**Figure S6.** **Greenland sleeper shark, turkey, and western terrestrial snake** **α-syn 1-25 fragments exhibit alpha-helix secondary structure as demonstrated by circular dichroism.** Random coils are observed in the human, Abingdon Island giant tortoise, Amur tiger, and common wall lizard.


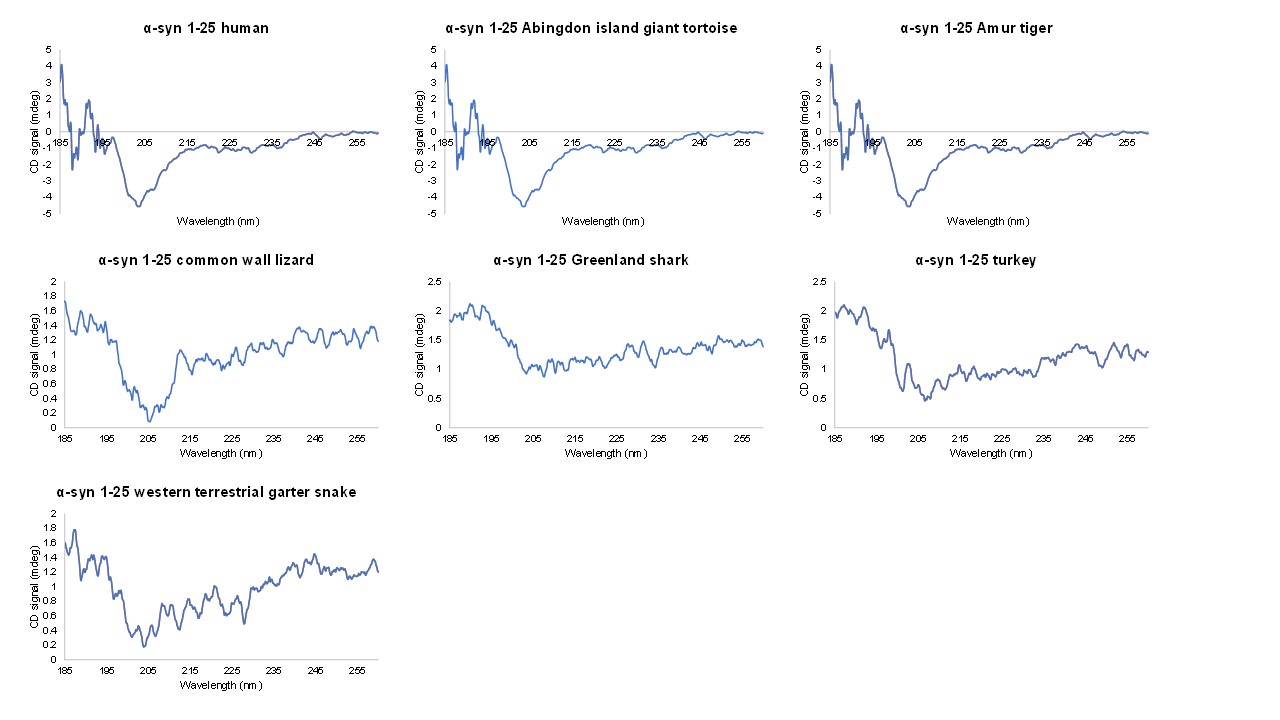


**Figure S7.** **Human, Abingdon Island giant tortoise, Amur tiger, common wall lizard, Greenland sleeper shark, turkey, and western terrestrial garter** **α-syn 37-61 fragments exhibit a beta-plated sheet secondary structure as demonstrated by circular dichroism.** Alpha-helix with a lesser degree of beta-plated sheet is observed with the common wall lizard, Greenland sleeper shark, turkey, and western terrestrial garter snake.


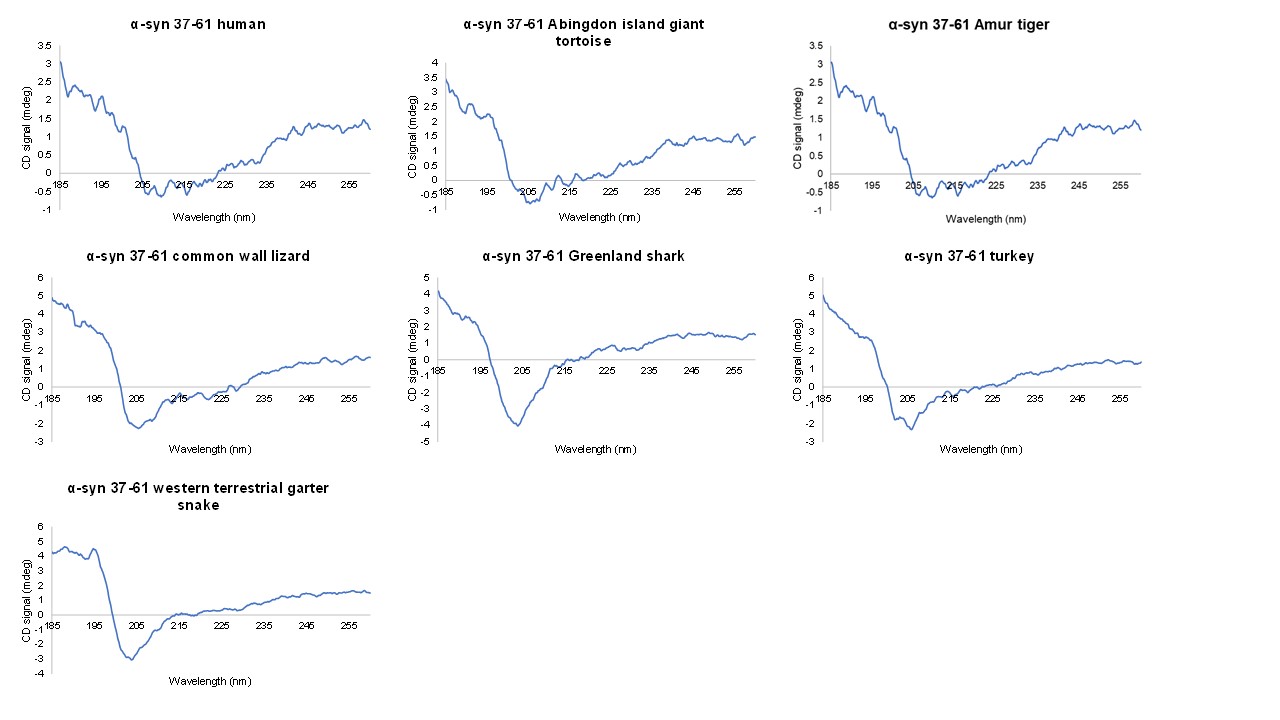


**Figure S8.** **Human, Abingdon Island giant tortoise, Amur tiger, common wall lizard, Greenland sleeper shark, turkey, and western terrestrial garter snake** **α-syn 62-86 fragments exhibit a beta-plated sheet secondary structure as demonstrated by circular dichroism.** Alpha-helix with a lesser degree of beta-plated sheet is observed with the common wall lizard. Prominent beta-plated sheets are noticed with the Greenland sleeper shark. A mixed structure of alpha-helix and beta-plated sheets are observed with the human, Abingdon Island giant tortoise, Amur tiger, common wall lizard, turkey, and western terrestrial garter snake.


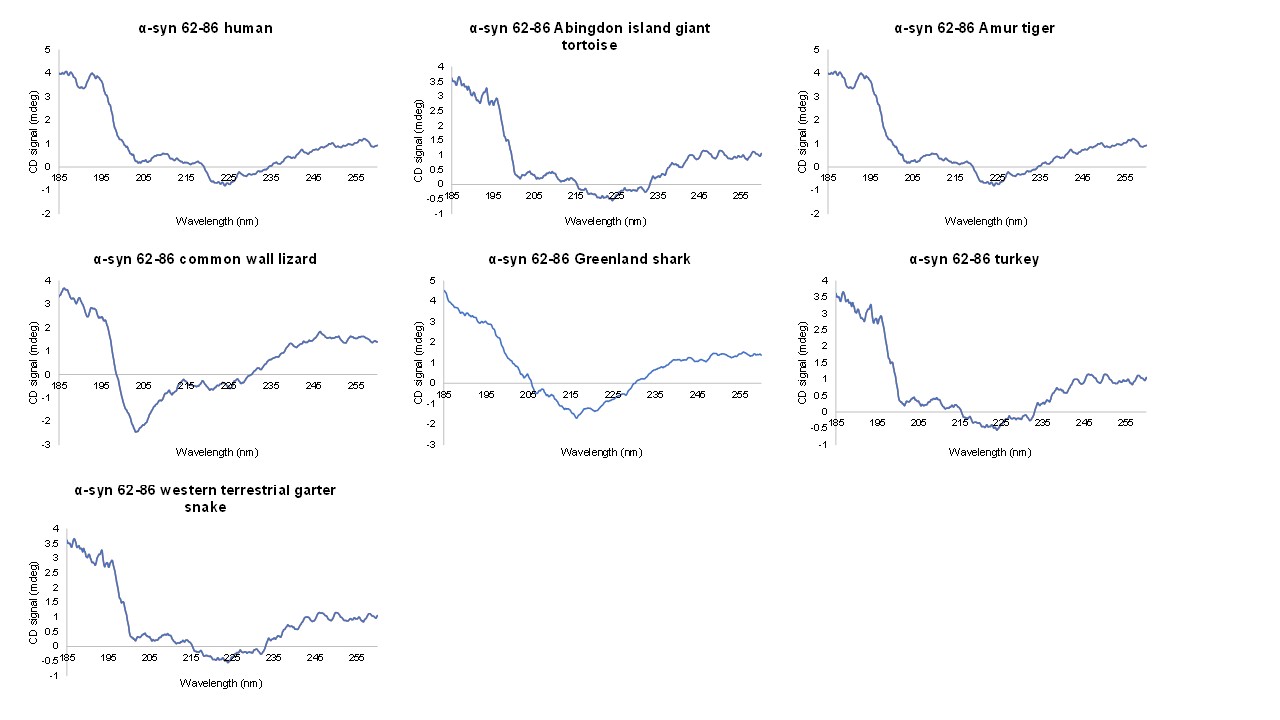

Supplement: Multimedia component 1 [file mmc1.docx]
